# Purinergic stimulation of K^+^-dependent Na^+^/Ca^2+^ exchanger isoform 4 requires dual activation by PKC and CaMKII

**DOI:** 10.1042/BSR20130099

**Published:** 2013-12-19

**Authors:** Xu Yang, Jonathan Lytton

**Affiliations:** *Libin Cardiovascular Institute of Alberta and Hotchkiss Brain Institute, Department of Biochemistry and Molecular Biology, University of Calgary, Calgary, Alberta T2N 4N1, Canada

**Keywords:** Ca^2+^-calmodulin-dependent kinase II (CaMKII), K^+^-dependent Na^+^/Ca^2+^-exchanger isoform 4 (NCKX4), P2Y-purinergic receptor, protein kinase C (PKC), AMPA, α-amino-3-hydroxy-5-methylisoxazole-4-propionic acid, [Ca^2+^]_i_, concentration of free ionized calcium, CaMKII, Ca^2+^/calmodulin-dependent protein kinase II, ER, endoplasmic reticulum, HEK-293, human embryonic kidney cell, HEK-293T, HEK-293 cells expressing the large T-antigen of SV40 (simian virus 40), NCKX, K^+^-dependent Na^+^/Ca^2+^-exchanger, NCX, Na^+^/Ca^2+^-exchanger, PDBu, phorbol 12,13-dibutyrate, PKA, protein kinase A, PKC, protein kinase C, PLC, phospholipase C, Tg, thapsigargin

## Abstract

K^+^-dependent Na^+^/Ca^2+^-exchanger isoform 4 (NCXK4) is one of the most broadly expressed members of the NCKX (K^+^-dependent Na^+^/Ca^2+^-exchanger) family. Recent data indicate that NCKX4 plays a critical role in controlling normal Ca^2+^ signal dynamics in olfactory and other neurons. Synaptic Ca^2+^ dynamics are modulated by purinergic regulation, mediated by ATP released from synaptic vesicles or from neighbouring glial cells. Previous studies have focused on modulation of Ca^2+^ entry pathways that initiate signalling. Here we have investigated purinergic regulation of NCKX4, a powerful extrusion pathway that assists in terminating Ca^2+^ signals. NCKX4 activity was stimulated by ATP through activation of the P2Y receptor signalling pathway. Stimulation required dual activation of PKC (protein kinase C) and CaMKII (Ca^2+^/calmodulin-dependent protein kinase II). Mutating T312, a putative PKC phosphorylation site on NCKX4, partially prevented purinergic stimulation. These data illustrate how purinergic regulation can shape the dynamics of Ca^2+^ signalling by activating a signal damping and termination pathway.

## Abstract 2

Activity of the K^+^-dependent Na^+^/Ca^2+^-exchanger, NCKX4, is stimulated by purinergic signals that depend on dual activation of two protein kinase pathways. This regulation provides a novel mechanism to shape Ca^2+^ signaling and thus to have important impact on neuronal processes.

## INTRODUCTION

A transient increase in intracellular Ca^2+^ {[Ca^2+^]_i_ (concentration of free ionized calcium)} is required for a wide range of signalling events, especially in excitable cells such as neurons. Since Ca^2+^ cannot be metabolized, an increase in [Ca^2+^]_i_ must be balanced by subsequent extrusion processes. In excitable cells where Ca^2+^ fluxes are high, this extrusion is predominantly mediated by Na^+^/Ca^2+^-exchangers, which have a high rate of transport [1,2]. In neuronal processes, exchanger-driven Ca^2+^ efflux has been shown to be due primarily to activity of members of the NCKX (K^+^-dependent Na^+^/Ca^2+^-exchanger) family of proteins [3,4].

The NCKX family consists of five members (NCKX1–5), each the product of a different *SLC24A* gene [5]. Structurally, the NCKX family shares a conserved topology that contains two clusters of transmembrane segments (each cluster consisting of five transmembrane spans) joined by a central cytoplasmic loop of varying length. Sequence similarity among members is highest within the transmembrane spans and lowest in the cytoplasmic loops [6–8]. NCKX proteins are also more distantly related to the NCX (Na^+^/Ca^2+^-exchanger) (SLC8) family of Na^+^/Ca^2+^-exchangers, best exemplified by the cardiac exchanger, NCX1 [9]. The structure of a bacterial Na^+^/Ca^2+^-exchanger homologue was recently published, confirming previous topological models for NCKX proteins, and the location of the ion binding and transport sites in the membrane segments [10]. Within brain neurons, NCKX2, NCKX3 and NCKX4 are all expressed with different abundance in different regions [11]. Recent experiments confirm that NCKX4 plays a critical role maintaining Ca^2+^ homoeostasis underlying adaptation and termination in olfactory neurons [12], and in the normal production of tooth enamel [13].

Extensive studies on cardiac NCX1 have established a complex array of regulatory processes that involve the central cytosolic loop of that protein and which depend on the binding of Ca^2+^, Na^+^, H^+^, anionic phospholipids and other factors [5,9]. Regulation among members of the NCKX family has not been studied in as much detail, but evidence has been presented indicating that Na^+^ occupancy of the transport sites can lead to time-dependent inhibition of function for NCKX2 [14], whereas studies using phorbol esters have established that PKC (protein kinase C) can stimulate NCKX2, but not NCKX3 or NCKX4 [15].

ATP, a purinergic agonist, has long been described as an important neurotransmitter and neuromodulator that regulates synaptic [Ca^2+^]_i_ dynamics [16,17]. ATP, released either from glial cells [18] or from synaptic vesicles [19], activates purinergic P2X and P2Y receptors. P2X receptors are ligand-gated ion channels, while P2Y receptors are G-protein-coupled receptors that signal through either cAMP/protein kinase A or inositol trisphosphate/DAG (diacylglycerol)/Ca^2+^ depending on the molecular sub-type of the receptor and the G protein it couples to: G_s_/G_i/o_ or G_q/11_ [20]. During neuronal stimulation, ATP and other excitatory neurotransmitters are co-released to exert co-modulation at the post-synaptic terminal, resulting in activation of a rapid ‘on’ switch that raises [Ca^2+^]_i_, and a slower ‘off’ process that is associated with Ca^2+^ extrusion to ensure proper [Ca^2+^]_i_ homoeostasis [19]. While many studies have focused on the influence that purinergic signalling has on Ca^2+^ entry processes that initiate signals [20–24], little is known about regulation of extrusion process that serve to dampen and/or terminate Ca^2+^ signals. Na^+^/Ca^2+^-exchangers, particularly those of the NCKX class, play an important role in the Ca^2+^ extrusion process [4,25,26]. While it seems probable that the efflux mediated by exchangers like NCKX4 would be regulated, such that activity is low during the early signal initiation phase and higher during the later termination phase, this has not been investigated previously. We hypothesized that purinergic signalling might be the mechanism providing such physiological regulation.

In the present study, we investigated the link between purinergic signalling and NCKX4 activity in transfected human embryonic kidney HEK-293T [HEK-293 cells expressing the large T-antigen of SV40 (simian virus 40)] cells, and found that NCKX4 activity can be stimulated by ATP through a P2Y-dependent signalling pathway. We demonstrated, using pharmacological interventions, that stimulation of NCKX4 requires the simultaneous activation of CaMKII (Ca^2+^/calmodulin-dependent protein kinase II) and PKC. Mutation of one candidate PKC site, T312, significantly reduced the extent of this stimulation.

## MATERIALS AND METHODS

### Materials

All chemicals and reagents were purchased from Sigma-Aldrich (sigmaaldrich.com) or EMD (emdcanada.com), and were analytical grade or higher, except where otherwise indicated. All molecular techniques were performed according to previously established protocols or manufacturer's instructions, unless otherwise specified.

### Cell culture and transfection

All cell culture reagents were obtained from Invitrogen (invitrogen.com). HEK-293 cells [HEK-293T)] were cultured in DMEM (Dulbecco's modified Eagle's medium) containing 10% (v/v) FBS, 1% (v/v) L-glutamine, 1% non-essential amino acids, and maintained in 5% (v/v) CO_2_ at 37°C. Transfection of Qiagen purified expression plasmid into HEK-293 cells was performed according to a previously published standard Ca^2+^-phosphate precipitation protocol [27]. Cells transfected with the parent expression vector, pcDNA3.1+ (Invitrogen), were used as negative controls.

### cDNA constructs and mutagenesis

Mouse NCKX4 cDNA in the vector pcDNA3.1(+) was used for all experiments described here. This construct was based on GenBank accession AY156046, and included a 72 nucleotide 5′-end extension, as present in GenBank accession AK44368, encoding an additional 17 amino acids at the N-terminus of the NCXK4 protein. In addition, the current clone included a silent C for G replacement at amino acid residue 44, and lacked the exon corresponding to the alternatively spliced region (amino acids 275–293). Site-directed mutagenesis was performed to create T256A, T312A, S325A, T256A/T312A and T256A/T312A/S325A mutants using the QuikChange II Site-Directed Mutagenesis Kit from Agilent Technologies (agilent.com), according to the manufacturer's protocol. Each NCKX4 mutant was sequenced to ensure only the intended change was present in the construct.

### Photometry and data analysis

Transfection and analysis of NCKX transport function was performed essentially as reported previously [27]. In brief, 2 days following transfection, HEK-293 cells grown on coverslips were loaded with 5 μM fura-2 AM (Invitrogen) and mounted in a perfusion chamber on the stage of an Olympus (olympuscanada.com) IX50 microscope, and observed using a 20X UAPO/340 0.75NA objective. A D-104 photometer (Photon Technology International; pti-nj.com)) was used to measure fluorescence emission at 510 nm after fura-2 excitation at 340 and 380 nm, collected from a field of ~50–100 cells, typically transfected with a frequency of 20–30%. The resulting 340/380 fura-2 ratio was calculated in FelixGX software (Photon Technology International). Unlike NCKX2, NCKX4 possesses a relatively high apparent affinity for K^+^ [27]. When NCKX4 was measured under a standard KCl concentration of 5 mM, activity levels were too rapid to be resolved as discrete rates in our system. Consequently, we developed the following protocol where [KCl] was rate-limiting (0.25 mM), thereby allowing precise resolution of NCKX4 activity. Perfusion solutions containing 10 mM Hepes-tetramethylammonium, pH 7.4, 10 mM D-glucose, 0.1 mM CaCl_2_ with either 145 mM NaCl (Na0K) or 144.75 mM LiCl and 0.25 mM KCl (Li0.25K) were used alternatively for 3 or 2 min intervals, respectively, at a rate of ~3 ml/min. The rates of Ca^2+^ transport during the reverse mode of NCKX4 operation (during Li0.25K perfusion) were determined using linear regression of the initial maximum linear rate of change of fura-2 ratio for each pulse. The rate for each pulse was normalized to that of the second pulse, and averages for each pulse and experimental condition, followed by two-way ANOVA and Bonferroni *post-test* comparing specific treatment conditions, were determined using GraphPad Prism 5.0.

## RESULTS

### Development of a functional assay for recombinant NCKX4

**Figure 1:**
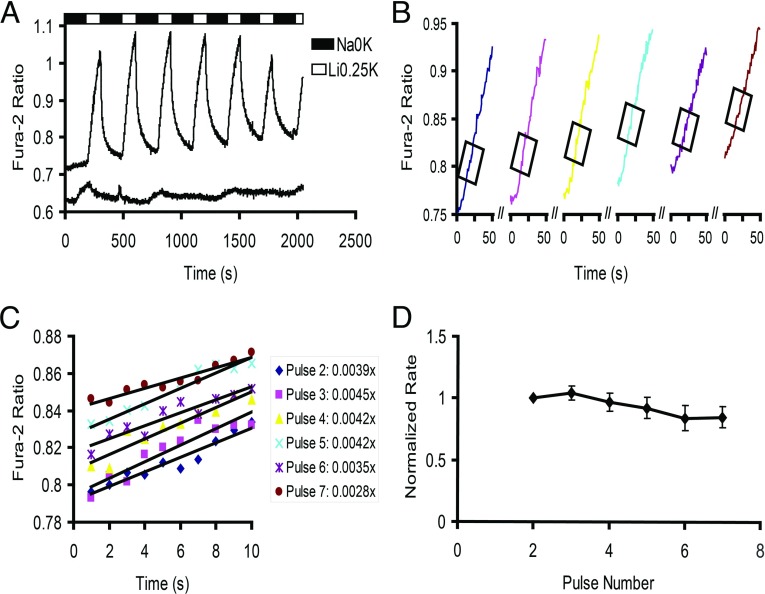
A quantitative assay for NCKX4 activity HEK-293 cells transfected with cDNA encoding mouse NCKX4, or vector alone, were loaded with fura-2, mounted in a perfusion chamber, and examined by microscopic photometry, as described in ‘Experimental Procedures’. (**A**) The fura-2 340/380 excitation ratio is plotted with time for either vector only-transfected cells (lower trace) or NCKX4-transfected cells (upper trace), both subjected to repeated perfusion switches between solution containing 145 mM NaCl (Na0K) or 144.75 mM LiCl and 0.25 mM KCl (Li0.25K). NCKX4 activity results in Ca^2+^ influx during the Li0.25K perfusion pulse, causing an increase in the fura-2 ratio. (**B**) The initial portion of each rising fura-2 ratio (pulses 2–7 from panel A) is shown expanded, with the steepest segment chosen for linear regression analysis boxed. (**C**) Individual data points and the linear regression fit for data from the boxed regions of panel (**B**), with the resulting slope values indicated. (**D**) The rates of fura-2 ratio rise (average±S.E.M.), internally normalized within each experiment to the rate of pulse 2, are shown averaged from four independent experiments.

HEK-293 cells grown on coverslips were transfected with recombinant constructs expressing mouse NCKX4 or vector alone as a negative control. Transfected cells loaded with fura-2 AM were subsequently mounted in a perfusion chamber on the microscope stage. When the perfusion solution containing 145 mM Na^+^, but no K^+^ (Na0K) was switched to one containing 144.75 mM Li^+^, 0.25 mM K^+^, but no Na^+^ (Li0.25K), a rapid increase in fura-2 ratio was observed in NCKX4-transfected HEK-293 cells but not in negative control (Figure 1A). The increase in fura-2 ratio also required the presence of K^+^ ions in the perfusion solution. Hence, this increase in fura-2 ratio represented Ca^2+^ influx mediated by reverse-mode NCKX4 activity. Data collected over repeated alternating perfusion switches between Na0K and Li0.25K for a total of six or seven pulses were then used to generate fura-2 versus time graphs in which the maximal initial rate of each individual pulse was determined based on linear regression analysis over a time frame of 10 s (Figures 1B and 1C). At noted above, the increase in fura-2 ratio was seen only when cells were transfected with constructs expressing NCKX4 (see Figure 1A). Furthermore, the initial rate of perfusion-induced fura-2 ratio increase was significantly greater than the subsequent rate of perfusion-induced decline at comparable fura-2 ratio values (Figure 1A). Thus, it can be concluded that these initial rates of fura-2 ratio increase corresponded to the rate of NCKX4-mediated Ca^2+^ influx at the time of the perfusion pulse. In all experiments, the rate for the first pulse was inconsistent with the subsequent pulses (although we have no explanation for this phenomenon, it is routinely observed in our laboratory [27]), and was therefore excluded from the analyses, and instead pulse two was used for internal rate normalization. Measured in this way, NCKX4 activity under control conditions was relatively stable with time and with respect to repeated perfusion pulses (Figure 1D).

### Purinergic agonist treatment results in stimulation of NCKX4 activity

**Figure 2:**
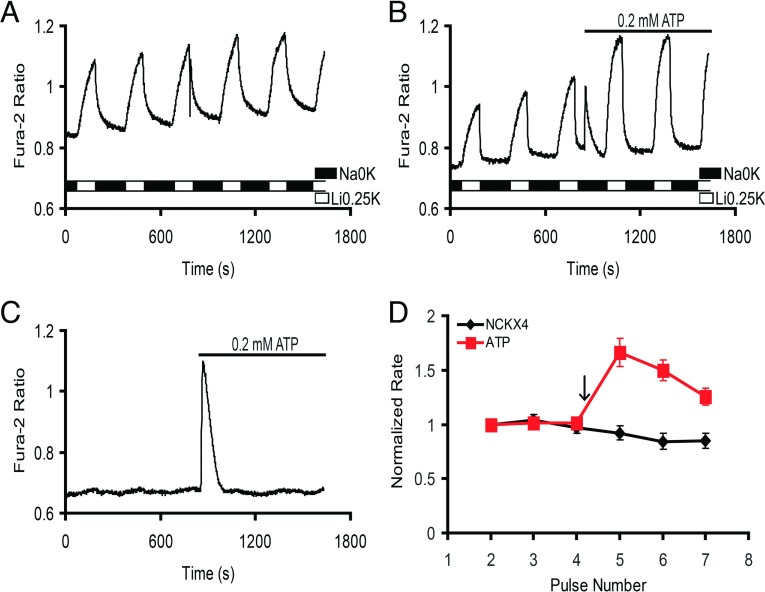
ATP treatment stimulates NCKX4 activity HEK-293 cells transfected with cDNA encoding mouse NCKX4 were analysed as described in Figure 1. (**A**) A time-control trace illustrating the repeated activation protocol to measure NCKX4 activity. (**B**) Following pulse four, cells were perfused with solutions containing 0.2 mM ATP as illustrated. (**C**) HEK-293 cells transfected with vector only, subjected to the same alternating perfusion switches and ATP treatment seen in panel (**B**). (**D**) Normalized NCKX4 activity (rate of fura-2 rise) is averaged (±S.E.M.) for one independent trial from each of four different experiments (control; black diamonds) or two independent trials from each of four different experiments (ATP, added at the arrow; red squares). Two-way ANOVA analysis revealed statistically significant differences in normalized rates between control and ATP-treated cells (*P*<0.001; 0.001; 0.05 for pulses 5–7, respectively). The magnitude of the increase in NCKX4 activity induced by ATP was relatively consistent among different samples from one experiment, but ranged from 1.8- to 4-fold between different experiments over the duration of this study.

To ascertain if purinergic agonists such as ATP exert an influence on NCKX4 activity, four perfusion pulses were used to establish the baseline NCKX4 activity. At that point, the alternating perfusion solutions were switched to ones containing 0.2 mM ATP. Time-based control experiments in the absence of ATP were also conducted (Figure 2A). Shortly after introducing ATP into the system, a large Ca^2+^ transient was observed, which quickly returned to base level (Figure 2B). The first NCKX4-activiting pulse following ATP addition displayed a dramatic increase in the magnitude of the fura-2 ratio compared with the baseline pulses, a change that gradually declined over subsequent pulses. The rate analysis of this set of experiments showed that on average, there was a 1.8-fold increase in NCKX4 activity following ATP stimulation (Figure 2D). ATP treatment of negative control cells (transfected with vector alone) elicited a large Ca^2+^ transient, but no Ca^2+^ changes in response to perfusion switches (Figure 2C). Hence, during the perfusion conditions employed in this experiment to measure NCKX4 activity in reverse, Ca^2+^-entry, mode, ATP treatment did not activate plasma membrane Ca^2+^ flux pathways other than NCKX4. Collectively, these data demonstrate that treatment with ATP stimulates NCKX4 activity. The magnitude of the stimulation induced by ATP was quite consistent from coverslip to coverslip within any given experiment, but varied significantly from day to day. Consequently, positive control experiments with ATP stimulation were performed in parallel with other treatments in all cases described below. Over the course of all the experiments described here, ATP stimulation resulted in increases in NCXK4 activity that ranged from 1.8- to 4-fold. It is also important to note that the absolute rates of fura-2 increase, while relatively consistent, varied significantly from coverslip to coverslip and from experiment to experiment. This variation is accommodated by normalizing the data to the baseline rate on each individual coverslip for all experiments.

**Figure 3:**
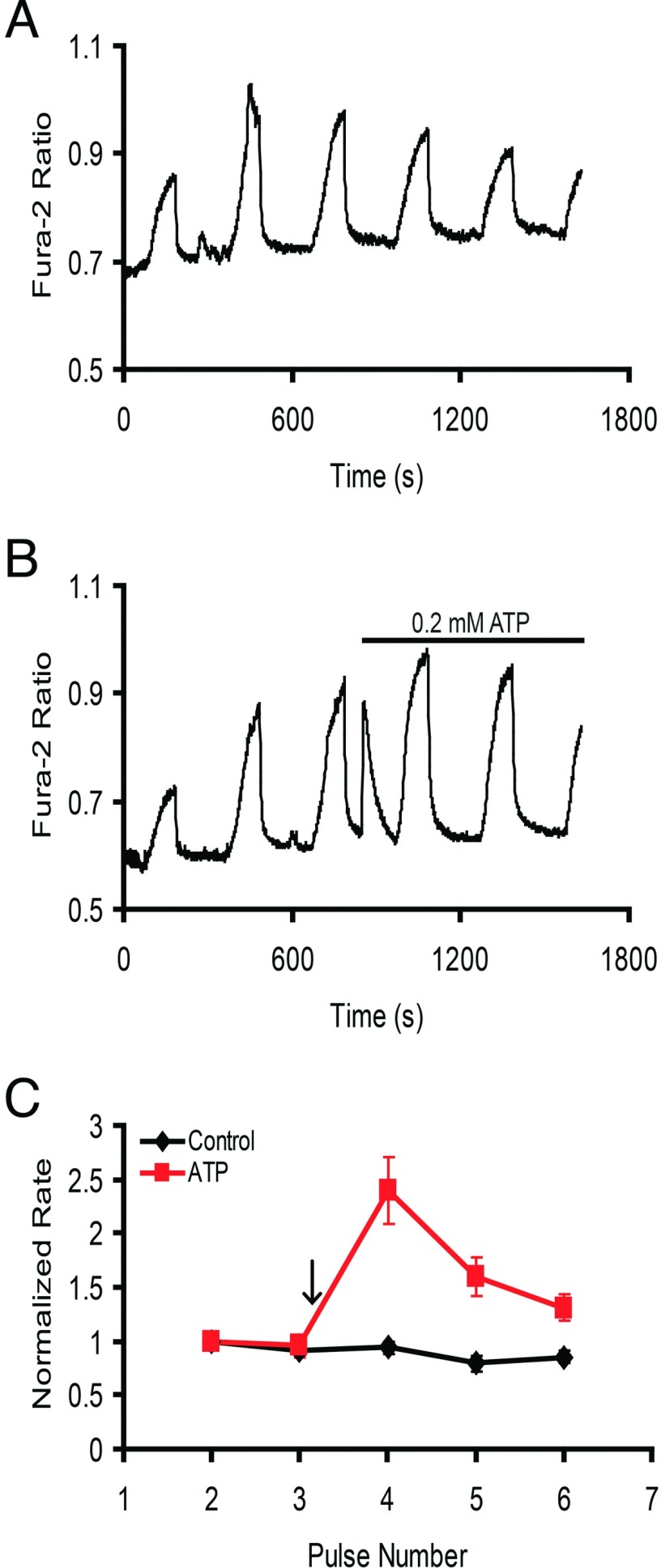
ATP stimulation of NCKX4 when [Ca^2+^] is rate-limiting HEK-293 cells transfected with cDNA encoding mouse NCKX4 were treated and analysed as described in Figure 2. All perfusion solutions contained 0.5 mM EGTA and 0.45 mM CaCl_2_ (to generate a free [Ca^2+^] of ~0.3 μM), and NCKX4 activity was induced by a change from 145 mM NaCl to 140 mM LiCl plus 5 mM KCl. (**A**) Time control example trace. (**B**) ATP stimulation example trace. (**C**) Averaged data from six independent experiments. Two-way ANOVA analysis revealed statistically significant differences in normalized rates between control and ATP-treated cells (*P*<0.001; 0.01 for pulses 4 and 5, respectively).

The assay employed to measure NCKX4 used K^+^ at a rate-limiting concentration of 0.25 mM. Therefore it was unclear if ATP stimulation increased the *V*_max_ of NCKX4 or increased the apparent affinity for K^+^. This issue was addressed by altering our assay protocol, so that Ca^2+^ was held at the rate-limiting concentration of 0.3 μM with an EGTA buffering system, while KCl was included at 5 mM. Under these conditions, ATP still resulted in a similar stimulation of NCKX4 activity (Figure 3). These data, in which ATP induced similar stimulation of NCKX4 activity when either K^+^ or Ca^2+^ was rate-limiting, suggest an effect on the *V*_max_ of transport activity.

### Activation of the P2Y receptor signalling pathway is necessary for NCKX4 stimulation

**Figure 4:**
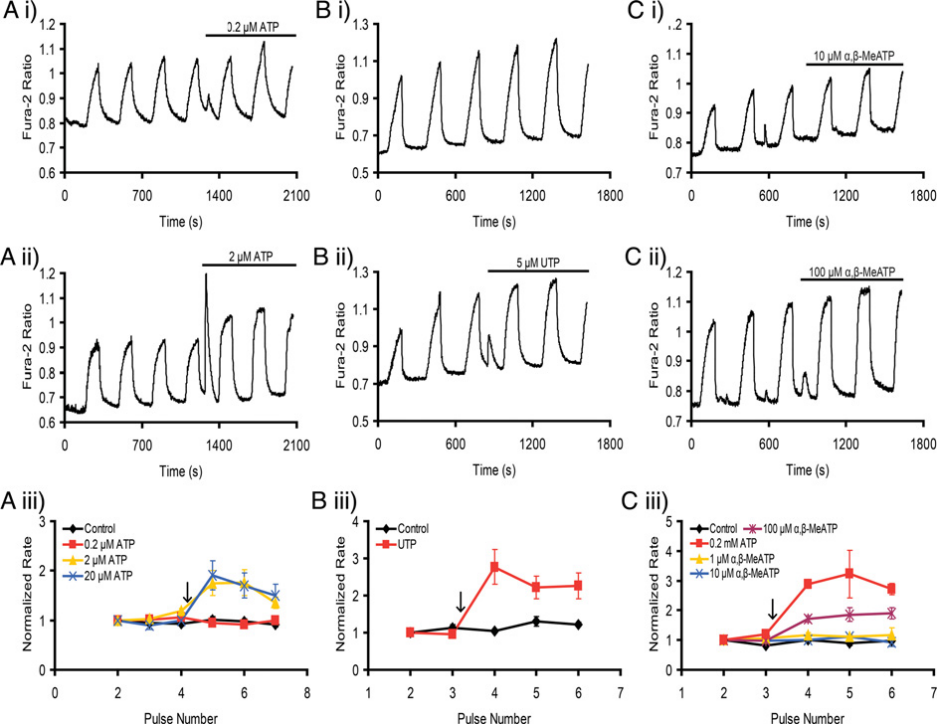
Examining purinergic specificity for stimulation of NCKX4 HEK-293 cells transfected with cDNA encoding mouse NCKX4 were analysed as described in Figure 2. (**A**) Cells were treated with different concentrations of ATP, as illustrated in the different panels. Control, *n*=4; 0.2 μM ATP, *n*=7; 2 μM ATP, *n*=3; 20 μM ATP, *n*=6. Addition of ATP is indicated by the arrow. Two-way ANOVA analysis revealed statistically significant differences in normalized rates between control and 2 μM (*P*<0.05 for pulses 4 and 5) or 20 μM ATP-treated cells (*P*<0.001; 0.01; 0.05 for pulses 4–6, respectively), but not 0.2 μM ATP-treated cells. (**B**) Cells were treated with (ii) or without (i) 5 μM UTP. Control, *n*=2; UTP, *n*=6. Addition of 5 μM UTP is indicated by the arrow. Two-way ANOVA analysis revealed statistically significant differences in normalized rates between control and UTP-treated cells (*P*<0.05 for pulses 4–6). (**C**) Cells were treated with different concentrations of the P2X-selective agonist, α,β-methylene ATP (α,β-MeATP) as illustrated. Control, *n*=3; 0.2 mM ATP, *n*=2; 1 μM α,β-MeATP, *n*=4; 10 μM α,β-MeATP, *n*=6; 100 μM α,β-MeATP, *n*=5. Addition of ligands is indicated by the arrow. Two-way ANOVA analysis revealed statistically significant differences in normalized rates between control and 0.2 mM ATP- (*P*<0.001 for pulses 4–6) or 100 μM α,β-MeATP-treated cells (*P*<0.01; 0.001; 0.001 for pulses 4–6, respectively) but not 1 μM or 10 μM α,β-MeATP-treated cells.

To examine which purinergic receptor pathway (P2X or P2Y) was activated by ATP to stimulate NCKX4, we first employed selective agonists. ATP is thought to activate P2Y (seven transmembrane-span G-protein-coupled) receptors at sub-micromolar concentrations, whereas much higher ATP levels are needed to activate P2X (ligand-gated ion channel) receptors [20]. Thus, NCKX4-transfected cells were treated with a range of ATP concentrations (Figure 4A). While the maximum stimulation was observed already at 2 μM ATP, 0.2 μM did not induce stimulation of NCXK4 activity. However, the Ca^2+^ transient typically observed upon ATP treatment of cells was much lower with 0.2 μM treatment than the response seen with higher concentrations of ATP, suggesting that under the conditions employed here, low concentrations of ATP do not efficiently activate P2Y receptors. Therefore this treatment regime was not able to distinguish P2X from P2Y receptor function. Consequently, NCKX4 stimulation was tested with 5 μM UTP, a concentration thought to activate P2Y but not P2X receptors [28,29]. This treatment induced a significant stimulation, suggesting a requirement for P2Y receptor activity in the NCKX4 response (Figure 4B). Finally, the treatment with α,β-methylene ATP, considered to be a P2X-specific agonist [30], was tested. Figure 4(C) shows that at concentrations below 100 μM, α,β-methylene ATP did not stimulate NCKX4. At 100 μM, however, α,β-methylene ATP partially stimulated NCKX4 but also caused a Ca^2+^ transient that resembled the effect of ATP. This suggested that at such a high concentration, α,β-methylene ATP lost specificity for P2X receptors. On balance, while not conclusive, these data support a role for P2Y, but not for P2X, receptors in the stimulation of NCKX4 activity.

**Figure 5:**
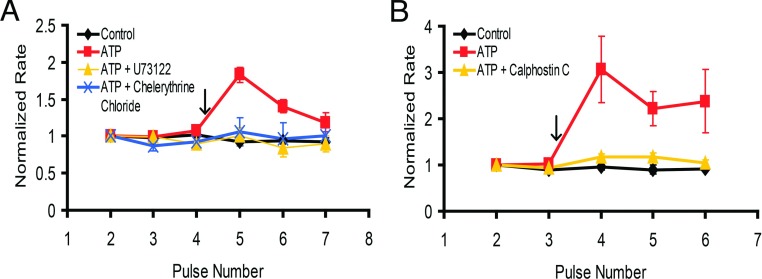
Inhibition of PLC or PKC prevents NCKX4 stimulation HEK-293 cells transfected with cDNA encoding mouse NCKX4 were stimulated with either 2 μM (panel A) or 0.2 mM ATP (panel B), added at the arrow, and analysed as described in Figure 2. Prior to initiation of the experiments, cells were pre-treated for 15 min with either 5 μM PLC inhibitor U73122, or the PKC inhibitors, chelerythrine chloride (5 μM) or calphostin C (100 nM), as indicated. (**A**) Control, *n*=4; ATP, *n*=5; ATP+U73122, *n*=4; ATP+chelerythrine chloride, *n*=4. Two-way ANOVA analysis revealed statistically significant differences in normalized rates between ATP-stimulated cells and cells pretreated with U73122 or chelerythrine chloride prior to ATP stimulation (U73122: *P*<0.001; 0.01 for pulses 5 and 6, respectively; chelerythrine chloride: *P*<0.001; 0.05 for pulses 5 and 6, respectively). (**B**) Control, *n*=3; ATP, *n*=4; ATP+calphostin C, *n*=8. Two-way ANOVA analysis revealed statistically significant differences in normalized rates between ATP-stimulated cells and cells pretreated with calphostin C prior to ATP stimulation (*P*<0.001; 0.05; 0.01 for pulses 4–6, respectively).

Since the results with selective purinergic agonists were ambiguous, we decided to interrogate the downstream signalling elements in the P2Y pathway using pharmacological interventions. P2Y receptors typically signal through the PLCγ (phospholipase C γ)–IP_3_–PKC pathway. If NCKX4 stimulation depends on activation of this pathway, interfering with any of the signalling components should prevent or inhibit exchanger stimulation. Therefore fura-2-loaded NCKX4-transfected cells were pre-incubated for 15 min in bathing solution that contained either a PLC inhibitor (U73122) or broad range PKC inhibitors (chelerythrine chloride or calphostin C) and subsequently tested for ATP-induced stimulation. Figure 5 shows that inhibition of either PLC or PKC abolished the stimulation following ATP treatment, indicating that ATP requires (likely via P2Y receptor signals) the activation of PLCγ and subsequently PKC to stimulate NCKX4.

### PKC activation alone is not sufficient for NCKX4 stimulation

**Figure 6:**
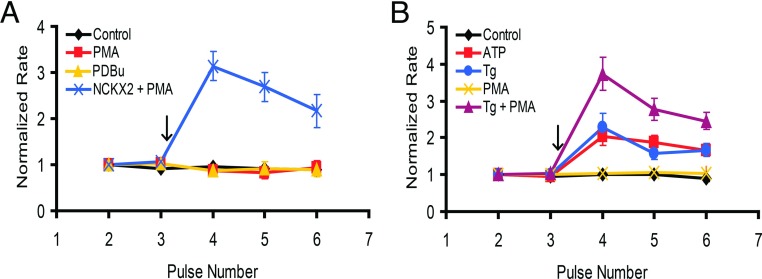
The effect of phorbol esters on NCKX4 activity Transfected HEK-293 cells were treated with various reagents, added at the arrow and maintained in all solutions thereafter, and analysed as described in Figure 2. (**A**) Cells were transfected with cDNA encoding NCKX4 and treated either with vehicle alone (control; *n*=3), 100 nM PMA (*n*=5), 100 nM PDBu (*n*=4), or NCKX2-transfected cells were treated with 100 nM PMA (*n*=2). Two-way ANOVA analysis revealed statistically significant differences in the normalized rates between control and PMA-treated NCKX2-transfected cells (*P*<0.001; 0.001; 0.01 for pulses 4–6, respectively), but not in PMA- or PDBu-treated NCKX4-transfected cells. (**B**) Cells were transfected with cDNA encoding NCKX4 and treated either with vehicle alone (control; *n*=5), 0.2 mM ATP (*n*=6), 0.5 μM Tg (*n*=14), 100 nM PMA (*n*=3) or Tg+PMA (*n*=12). Two-way ANOVA analysis revealed statistically significant differences in normalized rates between control and ATP- (*P*<0.001 for pulses 4–6), Tg- (*P*<0.001; 0.05 for pulses 4 and 6, respectively) or Tg+PMA-treated cells (*P*<0.001 for pulses 4–6), but not PMA-treated cells. Statistically significant differences were also found between ATP- and Tg+PMA-treated cells (*P*<0.001; 0.05 for pulses 4 and 5, respectively), and between Tg- and Tg+PMA-treated (*P*<0.001; 0.01 for pulses 4 and 5, respectively).

Since inhibitors of PKC prevented the stimulation of NCKX4, we next examined whether direct activation of PKC was sufficient for NCKX4 stimulation. However, neither phorbol esters PMA nor PDBu (phorbol 12,13-dibutyrate) at 100 nM were able to stimulate NCKX4, in sharp contrast to the robust stimulation observed for NCKX2, used as a positive control (Figure 6A). This stimulatory effect of phorbol ester alone on NCKX2 but not NCKX4 activity is consistent with our earlier studies [15]. We considered two possible explanations for the lack of phorbol ester-induced stimulation of NCXK4. First, it is possible that a ‘classical’ PKC isoform is necessary for stimulation of NCKX4, which under the conditions of our experiments cannot be activated by phorbol ester alone without a concomitant increase in [Ca^2+^] (NCKX2 stimulation induced with PMA has been shown to depend on PKC-ε, a ‘novel’ non-Ca^2+^-dependent isoform [15]). However, previously published work suggested that PMA alone is sufficient to activate even ‘classical’ PKC isoforms [31,32]. Thus, an alternative possibility is that stimulation of NCKX4 requires a second event in addition to PKC activation, possibly involving a Ca^2+^ signal. To test these ideas, NCKX4-transfected cells were treated with PMA and Tg (thapsigargin), an inhibitor of the SERCA (sarcoplasmic/endoplasmic reticulum Ca^2+^-ATPase) [33]. As shown in Figure 6(B), a robust stimulation of NCKX4 activity, greater even in magnitude than the stimulation seen with ATP treatment, was observed when Tg and PMA were applied together, but not with PMA alone. Interestingly, Tg alone was able to induce a significant change in the rate of NCKX4 transport, albeit not as large as that produced by Tg and PMA co-treatment. This result suggests a possible Ca^2+^-dependent second signalling event, in addition to PKC activation, required for NCXK4 stimulation.

### CaMKII activation is required for NCKX4 stimulation

**Figure 7:**
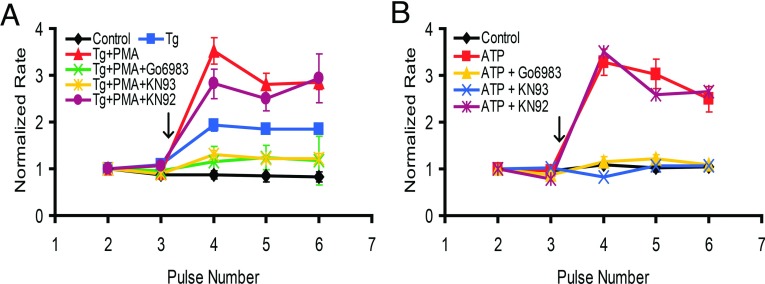
Inhibition of either classical PKC or CaMKII prevents NCKX4 stimulation HEK-293 cells transfected with cDNA encoding mouse NCKX4 were stimulated with either 0.5 μM Tg plus 100 nM PMA (panel A) or 0.2 mM ATP (panel B), added at the arrow, and analysed as described in Figure 2. Where indicated, cells were pretreated for 15 min prior to initiating the experiment with 100 nM Go6893 (classical PKC inhibitor), 1 μM KN93 (CaMKII inhibitor) or 1 μM KN92 (inactive control compound for KN93). (**A**) Control, *n*=4; Tg, *n*=7; PMA+Tg, *n*=8, PMA+Tg+KN93, *n*=6; PMA+Tg+KN92, *n*=3; PMA+Tg+Go6983, *n*=8. Two-way ANOVA analysis revealed statistically significant differences in normalized rates following Tg- or Tg+PMA-stimulation between untreated cells and cells pretreated with KN93 or Go6983 for pulses 4–6 (KN93: *P*<0.001; Go6983: *P*<0.001). (**B**) Control, *n*=3; ATP, *n*=5; ATP+Go6983, *n*=16; ATP+KN93, *n*=6; ATP+KN92, *n*=3. Two-way ANOVA analysis revealed statistically significant differences in normalized rates following ATP-stimulation between untreated cells and cells pretreated with Go6983 or KN93 for pulses 4–6 (*P*<0.001), but not for cells pretreated with KN92.

The fact that Tg, when used alone, was also able to partially stimulate NCKX4 suggested the involvement in regulation of a Ca^2+^-dependent pathway distinct from PKC activation. Ca^2+^ release from the ER, mediated by either IP_3_ or Tg, can trigger the activation of CaMKII. Hence, we carried out additional pharmacological intervention-based functional assays to evaluate this possibility. As shown in Figure 7(A), the CaMKII inhibitor, KN-93, but not its inactive congener, KN-92, effectively prevented Tg+PMA-induced NCKX4 stimulation. KN-93 was also effective in inhibiting ATP-induced stimulation of NCKX4 (Figure 7B). Furthermore, the ‘classical’ PKC inhibitor Go6983, was able to block stimulation of NCKX4 induced by Tg+PMA treatment, as well as stimulation by ATP (Figure 7). These data indicate that purinergic stimulation of NCKX4 requires activation of both PKC and CaMKII pathways, while blocking either PKC or CaMKII is sufficient to prevent purinergic stimulation.

### Mutagenesis of a putative PKC phosphorylation site in the central cytoplasmic loop of NCKX4 partially prevented purinergic agonist-mediated stimulation

**Figure 8:**
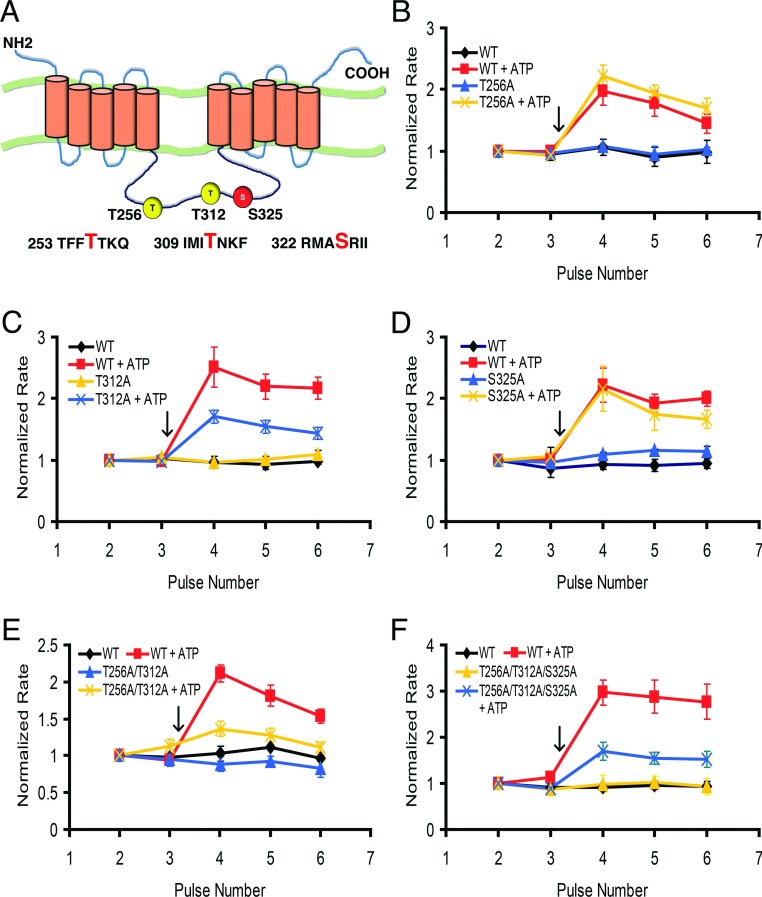
ATP stimulation of NCKX4 mutants HEK-293 cells were transfected with cDNA encoding either WT (wild-type) mouse NCKX4 or NCKX4 containing the indicated single, double or triple mutants. These cells were stimulated with 0.2 mM ATP, added at the arrow, and analysed as described in Figure 2. Two-way ANOVA analysis was used to test for statistical differences between normalized rates for mutants or treatments and pulses. (**A**) A cartoon topology model for NCKX4 illustrating the location of putative phosphorylation sites mutated in this study (T256, T312 and S325; the amino acid context of these residues is noted below the cartoon). (**B**) WT-control, *n*=4; T256A-control, *n*=2; WT+ATP, n=7; T256A+ATP, n=7. There was no statistically significant difference between WT+ATP and T256A+ATP. (**C**) WT-control, *n*=3; T312A-control, *n*=2; WT+ATP, *n*=9; T312A+ATP, *n*=17. The normalized rates for WT+ATP and T312A+ATP were statistically different (*P*<0.001; 0.01; 0.01 for pulses 4–6, respectively). (**D**) WT-control, *n*=3, S325A-control, *n*=2; WT+ATP, *n*=3; S325A+ATP, *n*=5. There was no statistically significant difference between WT+ATP and S325A+ATP. (**E**) WT-control, *n*=3; T256A/T312A-control, *n*=2; WT+ATP, *n*=10; T256A/T312A+ATP, *n*=12. The normalized rates for WT+ATP and T256A/T312A+ATP were statistically different (*P*<0.001; 0.001; 0.01 for pulses 4–6, respectively). (**F**) WT-control, *n*=3; T256A/T312A/S325A-control, *n*=2; WT+ATP, *n*=6; T256A/T312A/S325A+ATP, *n*=7. The normalized rates for WT+ATP and T256A/T312A/S325A+ATP were statistically different for pulses 4–6 (*P*<0.001).

Based on the idea that regulation of NCKX4 is associated with kinase modulation of specific regulatory sites, we screened NCKX4 for putative PKC phosphorylation sites using ‘(S/T)X(R/K)’ as the consensus sequence. Two potential candidates (T256 and T312) in the central cytoplasmic loop of the protein were identified (Figure 8A). An additional residue, S325, within a putative multi-phosphorylation motif for PKC, PKA (protein kinase A) and CaMKII, was also identified using a loose PKA consensus (‘(R/K)(R/K)XX(S/T)’ allowing one mismatch except for the (S/T) residue). We mutated each threonine or serine at these three sites to alanine (T256A, T312A and S325A) and evaluated the effect of ATP stimulation on the individual NCKX4 mutants. Figure 8 shows that T312A reduced the stimulatory effect of ATP by 50%, whereas the other two single mutants (T256A and S325A) were without effect. To examine if simultaneously mutating multiple phosphorylation sites could further prevent purinergic-induced enhancement of NCKX4, we created both a double (T256A/T312A) and a triple mutant (T256A/T312A/S325A). As illustrated in Figure 8, both of these double and triple mutants had a partial inhibitory effect on ATP-induced NCKX4 stimulation, similar to that observed for the T312A single mutant alone, suggesting no obvious additive suppression. Thus, these mutagenesis studies, while highlighting the contribution of T312, were not able to unambiguously identify putative phosphorylation sites solely responsible for purinergic stimulation of NCKX4. Further investigation is clearly needed to identify the target kinase site(s) required for full NCKX4 stimulation.

## DISCUSSION

In the present study, we examined the relationship between purinergic signalling and NCKX4 activity from a mechanistic perspective. We demonstrated through our functional assay using NCKX4-transfected HEK-293 cells that NCKX4 activity can be stimulated robustly with the purinergic agonist ATP. Based on pharmacological intervention and mutagenesis data, we propose a coincidence detection model in which the conditions for NCKX4 stimulation can only be fulfilled by the simultaneous activation of PKC and CaMKII. Purinergic agonists achieve this by activating P2Y receptors coupled via G_q/11_ protein to the activation of PLCγ, leading to the production of IP_3_ and DAG. IP_3_-triggered release of Ca^2+^ from the ER (endoplasmic reticulum) then binds to (i) calmodulin, which activates CaMKII, and (ii) PKC, which causes the kinase to translocate to the plasma membrane for subsequent activation by DAG. While our data support the P2Y receptor pathway as the predominant route for NCKX4 stimulation, the lack of a highly specific P2Y receptor antagonist and the ambiguous data using high concentrations of α,β-methylene ATP (Figure 4) prevented us from completely ruling out the involvement of the P2X receptor. Intriguingly, it appears that the Ca^2+^ which enters through NCKX4 during the induced bursts of reverse-mode operation is not sufficient, either alone or together with PMA treatment, to induce stimulation of NCKX4 activity, whereas Ca^2+^ release from the ER via Tg is sufficient (Figure 6). This suggests that Ca^2+^ released from the ER has preferential access to activation of CaMKII and/or PKC isoforms compared to Ca^2+^ entering across the plasma membrane, perhaps due to subcellular localization of these enzymes. This source-dependency for the effect of Ca^2+^ also argues against a role for P2X-mediated Ca^2+^ entry in the activation of NCKX4.

The dual requirement for both PKC and CaMKII activation to stimulate NCKX4 provides an explanation for why PMA treatment alone is insufficient for stimulation. However, despite previous studies to the contrary [31,32], it remains possible that PMA did not result in activation of the ‘classical’ PKC isoforms necessary for NCKX4 stimulation. To test the involvement of ‘classical’ PKC isoforms we used the selective inhibitor, Go6983, which prevented NCKX4 stimulation by either ATP or Tg+PMA (Figure 7). While these data suggest that one or more ‘classical’ PKC isoforms are involved, further experiments would be needed to confirm this supposition.

Given the dual requirement for PKC and CaMKII activation, it is surprising that Tg alone was sufficient for partial NCKX4 stimulation. Since the combination treatment of Tg and PMA results in a level of NCKX4 stimulation higher than that induced by ATP treatment, it is possible that Tg is activating a different, additive pathway. On the other hand, the fact that Tg+PMA stimulation can be prevented by treatment with either PKC or CaMKII inhibitors, just like ATP stimulation, suggests simply a more robust activation of similar pathways. Possibly the more sustained nature of Ca^2+^ release induced by Tg compared with ATP results in the production of lipid mediators, via regulation of phospholipase A2 or phospholipase D [34,35], that allow PKC as well as CaMKII activation under these conditions.

Although we have identified two kinase systems responsible for stimulation of NCKX4 activity, the target site for their actions remains elusive. Three sites conforming to either the PKC or CaMKII consensus and located in predicted cytoplasmic loops were tested by mutagenesis. Of these, only one (T312A) had a partial inhibitory effect on ATP-induced stimulation. It is possible that additional Ser or Thr residues that conform to a weaker consensus are required in addition to T312, and can account for kinase stimulation by direct phosphorylation of the NCKX4 protein. Alternatively, it is possible that other molecule(s) lying downstream from PKC and CaMKII are required for NCKX4 stimulation. Using selective pharmacological inhibition, we tested for the possible involvement of other kinases in the MEK [MAPK (mitogen-activated protein kinase)/ERK (extracellular-signal-regulated kinase) kinase], AMPK (AMP-activated protein kinase) and Akt (protein kinase B) families. None of these treatments, however, influenced ATP-induced stimulation of NCKX4 (results not shown).

The experiments in this paper all used ionic manipulation to induce reverse-mode Ca^2+^ entry to measure NCKX4 activity. This approach allows the isolation of NCKX activity from other plasma membrane or intracellular Ca^2+^ transport pathways. Since our data indicate an effect of stimulation on the maximal activity of the exchanger rather than individual ion-binding site affinities, it seems likely that purinergic stimulation will also influence the more physiologically relevant forward-mode, Ca^2+^ extrusion activity of the exchanger. Similar reasoning has been used when examining Ca^2+^-regulation of the cardiac exchanger, NCX1 [36].

Synaptic plasticity is crucially dependent on Ca^2+^-mediated kinase pathways modulating the activity of plasma membrane ion channels. Much work has demonstrated important roles for both PKC and CaMKII in the regulation of activity and membrane insertion of AMPA (α-amino-3-hydroxy-5-methylisoxazole-4-propionic acid)-receptor ion channels [37–39]. However, relatively little has been reported previously concerning the integrated regulation of Ca^2+^ homoeostasis that also requires control of Ca^2+^ efflux pathways. Here we have demonstrated that NCKX4 activity, a principle component of Ca^2+^ extrusion in brain neurons, is stimulated only by dual activation of PKC and CaMKII pathways. Intriguingly, the close family member, NCKX2, is stimulated similarly, but required only activation of PKC [15]. Thus, the precise control of Ca^2+^ homoeostasis depends on the cellular expression and subcellular localization of both transporter isoforms and regulatory kinase signalling pathways. The regulated activity of NCKX-mediated Ca^2+^ extrusion, integrated with Ca^2+^ entry pathways, is therefore critical to understanding molecular pathways of synaptic plasticity.

It remains unclear if NCKX4 stimulation corresponds to an allosteric increase in the activity of existing exchanger units in the plasma membrane, the recruitment of new units from a sub-membrane reservoir or both. In the context of Ca^2+^-mediated synaptic plasticity, regulation of the AMPA-receptor offers a potentially interesting comparison. In this case, stimulation of ion channel activity is mediated largely by recruitment of AMPA-receptor channels from an intracellular storage compartment to the post-synaptic membrane. Redistribution in response to synaptic Ca^2+^ signals requires the synergistic action of both CaMKII and PKC [40–42]. In addition, mutation of the key CaMKII site, S831 in the GluR1 subunit, reduced the magnitude of AMPA current stimulation, but did not prevent it [41,43]. These intriguing parallels between regulation of NCKX4 and the AMPA receptor clearly warrant further investigation.

In summary, our current study reveals a purinergic-dependent stimulation of NCKX4 that requires the simultaneous presence and activation of PKC and CaMKII. While the effect of stimulation can be reduced by mutating a putative PKC phosphorylation site T312, the specific mechanisms still require detailed investigation. At present, our data provide the first evidence of NCKX4 regulation in a physiologically relevant context, and open up the door to understanding the complex regulatory and physiological properties of this protein.
